# Recent Advances in the Synthesis and Applications of Nitrogen-Containing Macrocyclic Arenes

**DOI:** 10.3390/molecules30173646

**Published:** 2025-09-07

**Authors:** Jianhang Hu, Wanhua Wu, Cheng Yang

**Affiliations:** Key Laboratory of Green Chemistry & Technology of Ministry of Education, College of Chemistry, Sichuan University, 29 Wangjiang Road, Chengdu 610064, China

**Keywords:** macrocyclic arenes, nitrogen-containing macrocyclic arenes, mannich reactions

## Abstract

Macrocyclic arenes are rich-electron macrocycles bridged by methylene or methyl groups from aromatic rings substituted by hydroxyl or alkoxy groups. It has attracted great interest in host–guest chemistry and supramolecular self-assembly due to its clear cavity, adjustable structure and multifunctional binding ability. In particular, nitrogen-containing macrocyclic arenes including (hetero) aromatic moieties—constructed from building blocks such as pyrrole, carbazole, phenothiazine, and imidazole—have undergone rapid development, yielding a new generation of functional macrocycles, including calix[4]carbazoles, Tröger’s base-derived macrocycles, and phenothiazine-based architectures. These nitrogen-functionalized macrocycles feature rich chemical derivatization potential, unique structural and host–guest characteristics, and exceptional photophysical properties. They show great promise in molecular recognition, selective adsorption and separation, and the development of advanced functional materials. This review summarizes recent advances in the design, synthesis, and application of nitrogen-containing macrocyclic arenes, with a particular focus on structure–property relationships and emerging functions.

## 1. Introduction

Supramolecular chemistry studies molecular recognition and self-assembly processes governed by non-covalent interactions, encompassing a wide range of disciplines including chemistry, biology, and materials science [1,2,3]. Within this domain, macrocyclic hosts have served as essential structural scaffolds [4,5,6]. Since the seminal discovery of crown ethers, the design and synthesis of macrocyclic hosts have remained central to the advancement of this field [7]. Despite the extensive development of various macrocyclic frameworks [8,9,10,11,12,13,14,15], the exploration of novel macrocyclic systems continues to pose a formidable challenge. Macrocyclic arenes are rich-electron macrocycles bridged by methylene or methyl groups from aromatic rings substituted by hydroxyl or alkoxy groups [16,17,18]. These compounds typically feature electron-rich aromatic moieties connected via sp^3^-hybridized carbon atoms, conferring relatively rigid frameworks, unique electronic properties, and conformational dynamics. Recent advances in synthetic methodologies have enabled the construction of increasingly sophisticated macrocyclic arenes with well-defined geometries and functional potential [16,19,20,21,22,23,24]. Macrocyclic arenes are primarily prepared by macrocyclization with repeat aromatic units, such as phenyl/biphenyl-based derivatives (e.g., pillar[*n*]arenes, biphen[*n*]arenes, tiara[5]arenes) [25,26,27,28,29,30] and fused-ring compounds (e.g., prism[*n*]arenes, pagoda[*n*]arenes, naphthotubes) [31,32,33,34,35,36]. These macrocyclic hosts expand the research scope in supramolecular chemistry and provide new molecular platforms for the development of functional materials.

Compared to oxygen-containing analogs [16,37], nitrogen atoms exhibit superior functionalization versatility, enabling formation of amides, polysubstituted amines, quaternary ammonium salts, and N-oxides, while their variable valence states and stimulus responsiveness impart unique structural flexibility and functional diversity to macrocyclic arenes [38,39]. These characteristics establish nitrogen incorporation as a critical research focus in contemporary macrocyclic molecular engineering [40,41]. The most fundamental synthetic approach involves direct incorporation of nitrogenous units into the macrocyclic backbone. Friedel–Crafts alkylation efficiently links nitrogenous fragments to macrocyclic frameworks, while amide condensation routes show significant promise for ion-recognition applications, yielding macrocyclic arenes with distinctive peptide-bond architectures. Complementary strategies include Mannich condensation, transition metal-catalyzed couplings, coordination-directed assembly, and nucleophilic substitutions, each offering distinct features: Friedel–Crafts alkylation bridges electron-rich arenes; Mannich reactions require specific aromatic amine/carbonyl components; amide condensations usually employ amino acid derivatives; nucleophilic substitutions utilize halocarbon substrates. These synthetic advances enable macrocyclic arenes to demonstrate superior performance in molecular recognition, selective adsorption, and functional materials science. In addition, there are some methods for the synthesis of nitrogen-containing macrocyclic compounds [42,43], such as the synthesis of macrocyclic compounds containing 1,10-phenanthroline [44,45]. Asymmetric synthesis methods are also often used for nitrogen-containing compounds [46,47]. Finally, some macrocyclic compounds without catalysis [48,49,50,51] and light-controlled synthesis [52] also play an important role.

This review mainly summarizes the research progress of nitrogen-containing macrocyclic aromatic hydrocarbons in the past five years. Firstly, the synthesis strategies are classified according to the structural position of the nitrogen atom, and the system with nitrogen as the bridging site and the system with nitrogen in the non-bridging position are distinguished. It includes three types: Friedel–Crafts alkylation reaction, nucleophilic reaction, and Mannich reaction. Subsequently, the structural features and physicochemical properties of nitrogen-functionalized macrocycles are systematically compared with those of traditional phenol-based macrocycles. Finally, the current progress in their applications—particularly in molecular recognition, selective adsorption and separation, and the development of functional materials—is summarized. This review aims to offer both theoretical insight and practical guidance for the rational design of next-generation macrocyclic arene systems.

## 2. Synthesis of Nitrogen-Containing Macrocyclic Arenes

Despite substantial advances in macrocyclic arene research over the past decade, current synthetic systems predominantly utilize benzenoid cores with hydroxy/alkoxy substituents [16]. Research on extended π-systems (e.g., biphenyl, acene) remains underdeveloped, primarily hindered by availability of synthetic monomers, synthetic challenges, poor solubility, and reaction site selectivity issues. Nitrogenous compounds are pivotal in medicinal chemistry and functional materials, owing to structural diversity and facile functionalization. These properties render them ideal precursors for macrocycle construction, enabling development of structurally unique macrocyclic arenes. Based on nitrogen-atom positioning within the macrocyclic framework, these systems classify into two categories: (1) nitrogen is a bridging atom of aromatic subunits (e.g., Tröger’s base-functionalized macrocycles [53], phenothiazine-based macrocycles [54]); (2) nitrogen is not the bridging atom, but serves as a heteroatom within a heterocycle, such as calix[*n*]pyrroles [55,56], calix[*n*]carbazoles [57], cyclocarbazoles [58,59,60,61], phenothiazine derivatives [62], imidazole-based macrocycles [63], and indole-containing architectures. Macrocyclic arenes featuring bridging nitrogen atoms are often synthesized through aniline derivatives. These nitrogen centers exhibit versatile reactivity for further functionalization. Macrocyclic arenes having non-bridging nitrogen are typically constructed via Friedel–Crafts alkylation, which generally have component units of relatively large p plane and could show unique photophysical properties.

### 2.1. Macrocyclic Arenes with Non-Bridging Nitrogen

Certain macrocyclic arenes incorporate nitrogen atoms at non-bridging sites. Their synthetic approach closely resembles that of phenol-based macrocyclic arenes, involving one-pot condensation of electron-rich aromatics with paraformaldehyde catalyzed by Brønsted acids, particularly for constructing macrocycles comprising identical subunits.

**Figure 1:**
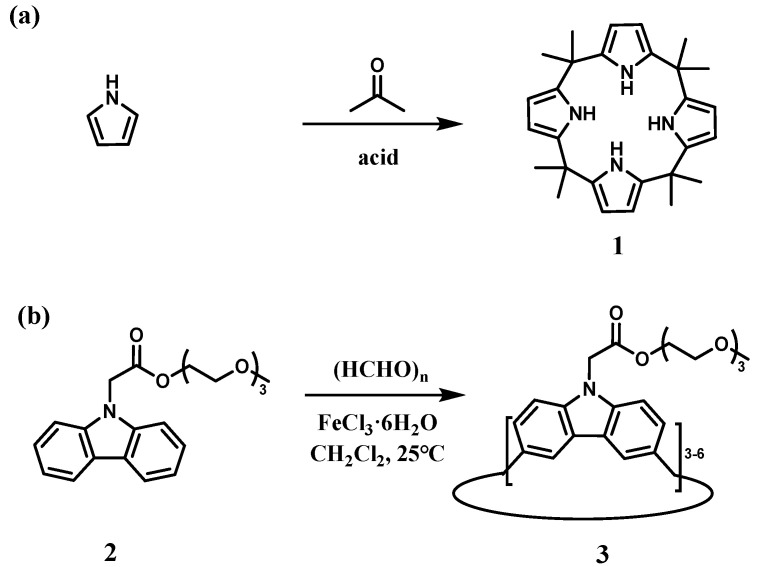
(**a**) Synthesis of calix[4]pyrrole **1**; (**b**) synthesis of calix[3]carbazole compounds [57]. Copyright © 2016, American Chemical Society.

Research on nitrogen-containing macrocycles originated in the late 19th century. In 1886, Baeyer and co-workers described the HCl-catalyzed condensation of pyrrole with acetone (Figure 1a) [64]. As a representative system, calix[4]pyrrole **1** mediates halide anion recognition via N−H···X hydrogen bonding, while simultaneously binding ion pairs through cation–π interactions [55]. Pioneered by the research groups of Sessler and co-workers, derivatives based on the calix[4]pyrrole scaffold have been extensively developed. Despite a century of research on calix[4]pyrrole, its straightforward synthesis, structural tunability, and superior host–guest properties sustain its prominence in modern supramolecular chemistry.

Yang and co-workers achieved a breakthrough in carbazole macrocycle synthesis, pioneering the methylene-bridged carbazole macrocycle (Figure 1b) [57]. Employing carbazole monomer **2** and paraformaldehyde as starting materials with FeCl_3_·6H_2_O as catalyst, they achieved one-step cyclization in CH_2_Cl_2_ at ambient temperature. This reaction afforded product **3**, predominantly comprising calix[3]carbazole (20% yield), with minor amounts of calix[4–6]carbazole byproducts.

**Figure 2:**
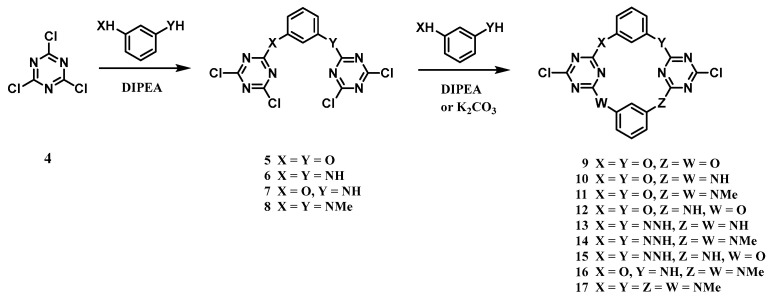
Synthesis of calix[2]arene[2]triazine macrocyclic **9**–**17** [65]. Copyright © 2004, American Chemical Society.

Wang and co-workers efficiently synthesized a series of novel nitrogen- or oxygen-bridged calix[2]arene[2]triazine macrocycles (Figure 2) [65]. Their stepwise approach involved the nucleophilic substitution of a triazine derivative **4** to form trimeric intermediates **5**–**8**, followed by a [3 + 1] cyclization that yielded macrocycles **9**–**17**. X-ray analysis confirmed a 1,3-alternating conformation in the solid state, with the bridging heteroatoms arranged coplanarly. The researchers found that these heteroatoms critically modulate the cavity geometries through electronic, conjugative, and steric effects, which, in turn, dictate distinct supramolecular assemblies. This work significantly expanded the known diversity of heteroatom-bridged calixarene macrocycles.

**Figure 3:**
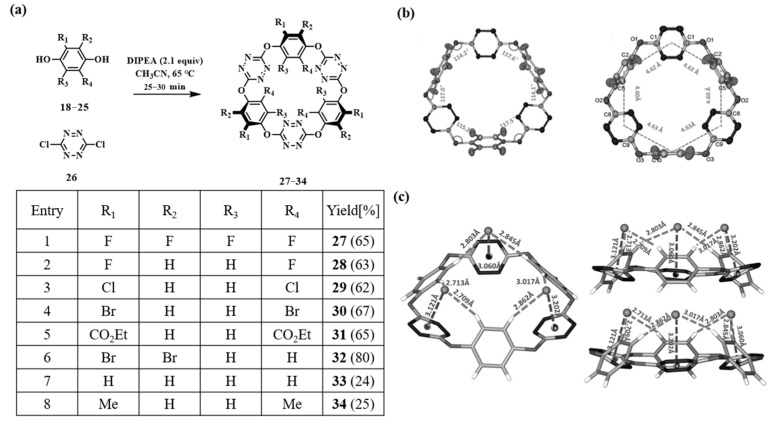
(**a**) The synthetic route of O6-corona[3]arene[3]tetrazines; (**b**) single-crystal structure of O_6_-corona[3]arene[3]tetrazines; (**c**) single-crystal structure of Cl^−^ complex with O_6_-corona[3]arene[3]tetrazines [66]. Copyright © 2014 WILEY-VCH Verlag GmbH&Co.KGaA, Weinheim.

The same group reported the synthesis of O_6_-corona[3]arene[3]tetrazine macrocycles via aromatic nucleophilic substitution (Figure 3) [66]. Macrocycles **27**–**34** (Figure 3a) were synthesized in one step through mild nucleophilic substitution between 1,4-dihydroxybenzene derivatives **18**–**25** and 3,6-dichlorotetrazine **26**, with yields ranging from 25% to 80%. Single-crystal analyses of **27** and **28** revealed hexagonal macrocycles featuring nearly planar arrangements of oxygen bridges and tetrazine rings (Figure 3b). Variable-temperature NMR spectroscopy indicated conformational changes between solid and solution states. Owing to their electron-deficient character, these macrocycles exhibit chloride binding via anion–π interactions, as demonstrated by **33** (Figure 3c). This study demonstrates that aromatic nucleophilic substitution is a viable strategy for macrocyclization, thereby broadening the structural scope of macrocyclic chemistry.

**Figure 4:**
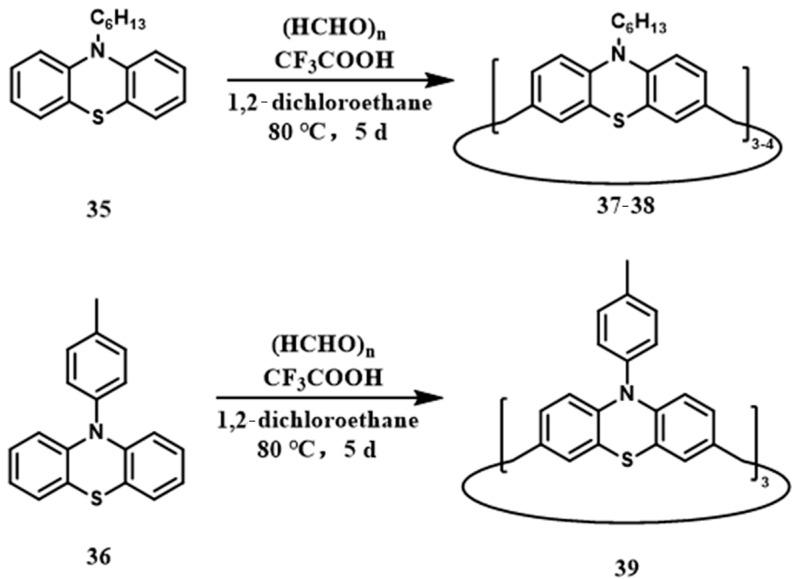
The synthetic route of macrocyclic **37**–**39**.

Esser and co-workers reported a novel methylene-bridged macrocycle derived from phenothiazine (Figure 4) [62]. The synthesis was carried out using N-substituted phenothiazine derivatives and paraformaldehyde in 1,2-dichloroethane, with trifluoroacetic acid as the catalyst at 80 °C for 120 h. When alkyl-substituted monomer **35** was employed, both calix[3]phenothiazine **37** (15% yield) and calix[4]phenothiazine **38** (4% yield) were obtained. In contrast, the p-methylphenyl-substituted monomer **36** selectively produced calix[3]phenothiazine **39** in 30% yield. X-ray crystallographic analysis confirmed a non-pyramidal AAB configuration for **39**. Quantum chemical calculations indicated that the cone conformation (AAA) is 1.2–1.3 kcal/mol higher in energy than the observed non-cone form.

**Figure 5:**
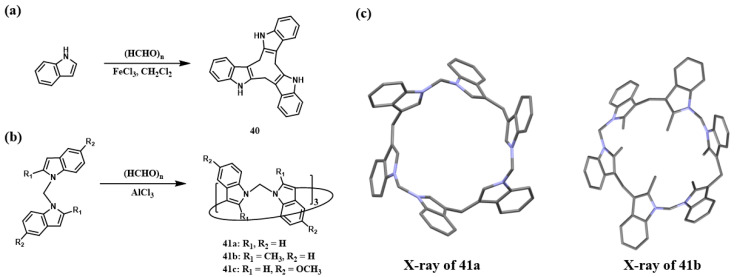
(**a**,**b**) Synthesis of indole[3]arene and bisindole[3]arenes; (**c**) crystal structures of **41a**–**41b** [67]. Copyright © 2021 Chinese Chemical Society.

Yang and co-workers reported bisindole[3]arenes—a novel class of indole-based macrocycles (Figure 5) [67]. The initial direct cyclization of indole with paraformaldehyde afforded the methylene-bridged cyclic trimer **40**, characterized by (i) a small cavity and (ii) methylene linkages at the 2′- and 3′-positions of the indole rings (Figure 5a). An optimized one-pot condensation of bisindole derivatives with paraformaldehyde in CH_2_Cl_2_, catalyzed by AlCl_3_ at room temperature, yielded target compounds **41a** (4.8%) and **41b** (10.3%) (Figure 5b). X-ray crystallographic analysis confirmed that all bisindole[3]arenes possess C_3v_ symmetry. Notably, **41a** displays alternating up/down orientations of the indole units relative to the macrocyclic plane, whereas the methyl-substituted **41b** adopts a petal-like conformation (Figure 5c).

**Figure 6:**
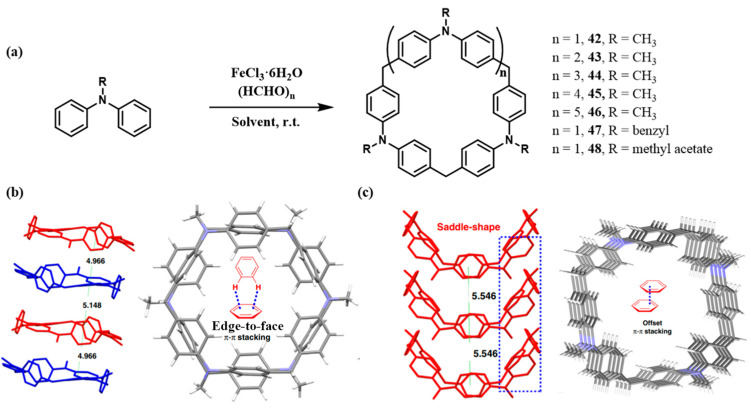
(**a**) Synthesis and structure diagram of DPA[n] macrocycle 42–48; (**b**) single-crystal stacking diagram of **42**; (**c**) single-crystal stacking diagram of **43** [68].

Shi and co-workers reported a series of DPA[n] macrocycles (**42**–**48**) derived from methyldiphenylamine units, representing a significant advancement in the synthesis and self-assembly of aniline-based macrocycles (Figure 6a) [68]. These compounds were efficiently synthesized via a one-pot procedure and exhibit structural features distinct from classical aniline derivatives. All benzene rings adopt either coplanar or perpendicular conformations. X-ray crystallographic analysis revealed that macrocycle **42** adopts a planar structure stabilized by intermolecular edge-to-face π–π interactions. In contrast, **43** displays a V-shaped conformation with offset face-to-face π–π stacking within its molecular assemblies (Figure 6b,c). This stacking mode facilitates hierarchical self-assembly: (i) **42** and **43** form columnar structures; (ii) van der Waals interactions at the benzene ring edges promote staggered layering; and (iii) ultimately, three-dimensional porous frameworks are formed.

**Figure 7:**
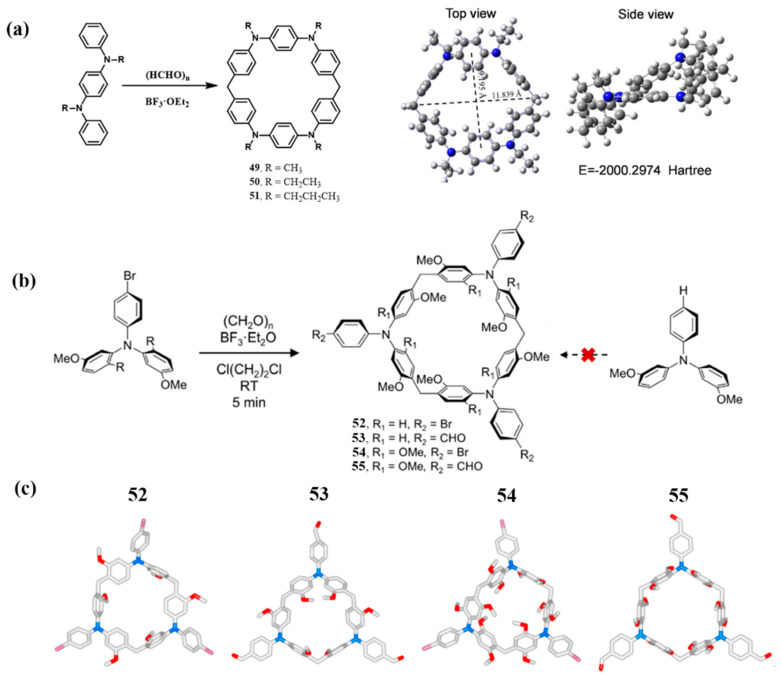
(**a**) Synthesis and structure of macrocycle **49**–**51** and single crystal of macrocycle **50**; (**b**) synthesis and structure diagram of **52**–**55**; (**c**) the single-crystal structure diagram of **52**−**55**. Copyright © 2024 Wiley-VCH GmbH.

Yang and co-workers synthesized novel aniline-based macrocycles **49**–**51** via a one-pot condensation of diphenylamine with paraformaldehyde (Figure 7a) [69]. Structural analysis revealed that An[2] comprises six benzene rings adopting distinct conformations, with four in coplanar orientation, forming a semi-open cavity of approximately 9 Å in diameter.

Building upon earlier studies of aniline macrocycles, significant progress has been made in the cyclization of triphenylamine derivatives. Zhao and co-workers employed bromotriphenylamine as a precursor to synthesize C_3_-symmetric TPA[3] macrocycles (Figure 7b,c). Brominated derivatives improved yields compared to conventional substrates, while halogen substituents facilitated further post-functionalization. The resulting macrocyclic framework consists of six benzene rings connected through alternating methylene and nitrogen bridges, forming a cavity (~10 Å) comparable in size to that of pillar[6]arene [70].

### 2.2. Macrocyclic Arenes with Nitrogen-Bridging Macrocycles

#### 2.2.1. Nucleophilic Reaction

**Figure 8:**
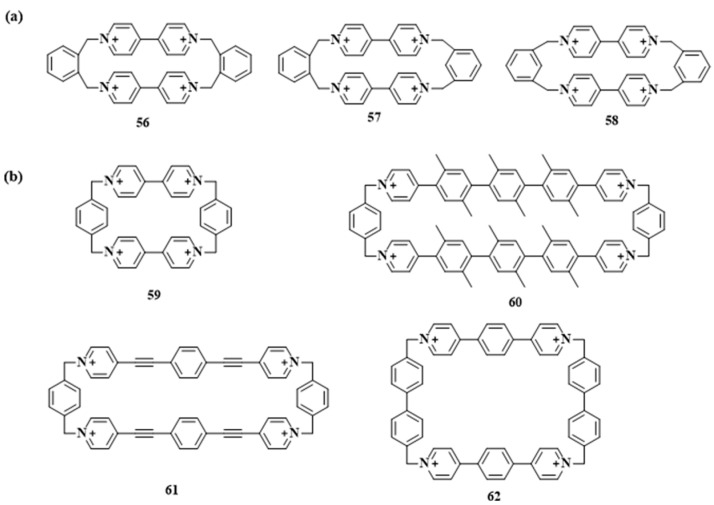
(**a**) The chemical structures of 4,4 ‘-bipyridine macrocycles **56**–**58**; (**b**) the chemical structure of bis(paraquat-*p*-phenylene) **59** and its derivatives macrocyclic **60**–**62**.

Research on paraquat-based macrocycles began in the 1980s. Hünig and co-workers synthesized paraquat macrocycles **56**–**62** (Figure 8a) [71], followed by systematic investigations of their radical cation properties [72]. Stoddart and co-workers prepared cyclobis(paraquat-p-phenylene) (CBPQT^4+^, **55**; Figure 8b), demonstrating its host–guest complexation with electron-rich species. This finding laid the foundation for the development of mechanically interlocked molecules. Despite over three decades of study, CBPQT^4+^ remains a focus of active research due to its accessible synthesis and unique electronic characteristics. Recent efforts have yielded structural derivatives (e.g., **56**–**58**), thereby broadening the scope of supramolecular applications.

**Figure 9:**
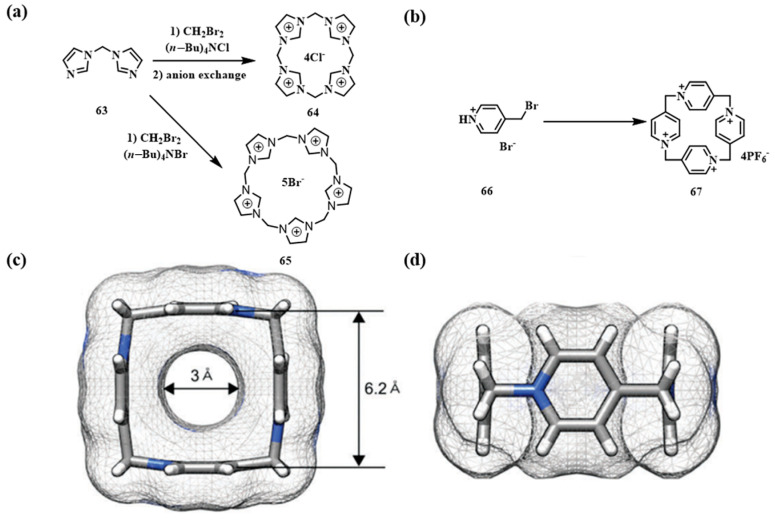
(**a**) Synthesis of calix[4]imidazoline **64** and calix[5]imidazoline **65**; (**b**) synthetic route of macrocyclic **67**; (**c**,**d**) single-crystal structure of **67**.

Chun and co-workers reported the first calixarene-like system derived from imidazolium salts, marking a significant advance in heterocyclic macrocycle chemistry (Figure 9a) [63]. Using methylene-bridged imidazole **63** as a precursor, they obtained calix[4]imidazolium **64** and calix[5]imidazolium **65** via nucleophilic substitution. This strategy overcomes the structural uniformity of traditional calixarenes, offering greater design versatility. Subsequently, Szumna and co-workers reported the synthesis of pillar[4]pyridinium macrocycles (Figure 9b), contributing to the development of pyridinium-based systems [73]. They employed pyridinium salt **66** as the precursor, achieving macrocycle **67** in 50% yield through an efficient one-step nucleophilic substitution. Notably, the choice of counterion significantly influenced the solubility of the resulting macrocycles. X-ray crystallographic analysis revealed that pillar[4]pyridinium **67** adopts a square, box-like conformation (side length: 6.2 Å; cavity diameter: 3 Å) (Figure 9c,d), a preorganized geometry well-suited for applications in molecular recognition and self-assembly.

**Figure 10:**
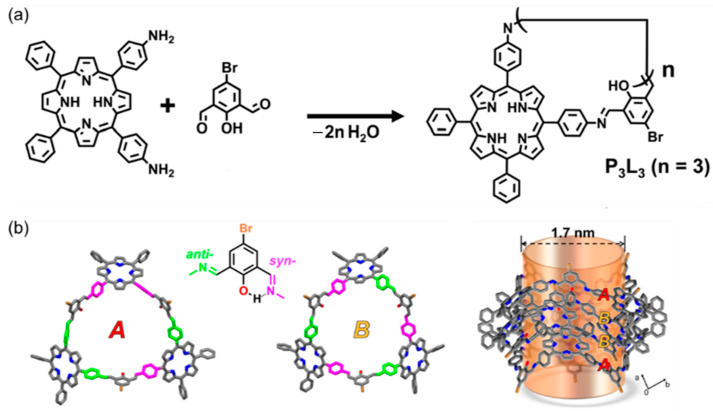
(**a**) The schematic diagram of the synthesis of imine porphyrin macrocycle P_3_L_3_; (**b**) conformation and stacking of P_3_L_3_ in the crystal structure.

Kim and co-workers synthesized triangular porphyrin macrocycle P_3_L_3_ by imine condensation reaction using cis-diaminophenyl porphyrin and isophthalaldehyde linker as building blocks (Figure 10a) [74]. P_3_L_3_ molecules have two conformations A and B with different cis–trans arrangements in the solid state, and stack with each other through π-π and CH-π interactions to form nanotubes with a diameter of 1.7 nm (Figure 10b). Gas adsorption experiments and powder X-ray diffraction analysis show that the pores in the P3L3 crystal are permanent and will not collapse due to the reversibility of the imine bond. By adjusting the angle of the bending connector, some columns of large ring structures with different sizes can also be obtained.

#### 2.2.2. Mannich-Type Macrocyclization

**Figure 11:**
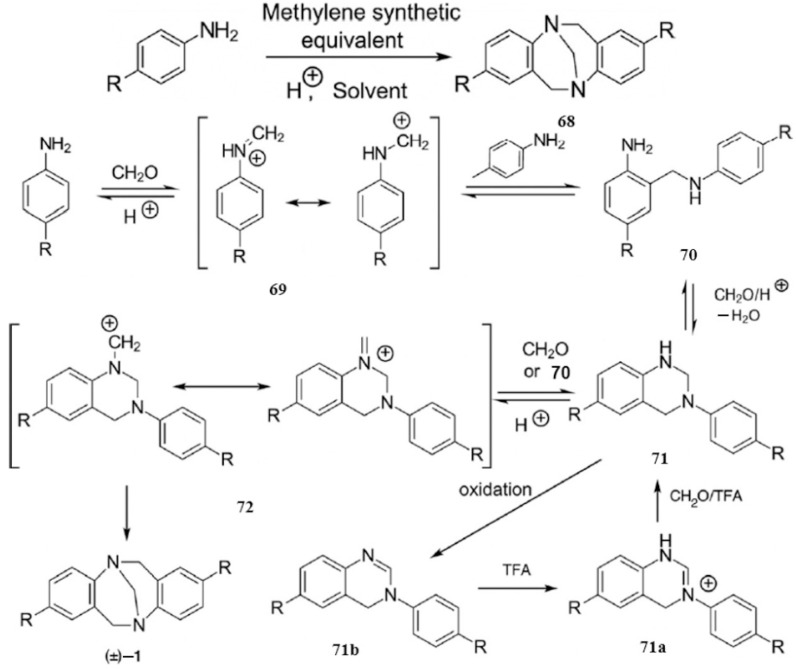
The possible reaction mechanism of TB proposed by Wagner and Farrar.

Tröger’s base (TB), a structurally distinctive class of organic compounds, was first reported by Carl Julius Ludwig Tröger in 1887 [75,76]. Research in this field has spanned nearly 140 years, with a notable resurgence over the past two decades—approximately 60% of all related publications have appeared during this period [77,78]. Structurally, TB features a characteristic V-shaped geometry defined by (1) methylene-bridged aromatic rings, (2) primary amine nitrogen atoms located at the V-shaped termini, and (3) a rigid, highly symmetric framework. This unique structure imparts three key properties: (i) intrinsic chirality, with enantiopure forms accessible via chiral HPLC resolution; (ii) utility as chiral building blocks for macrocyclic systems; and (iii) pronounced steric rigidity. The established synthetic route (Figure 11) follows a Mannich-type reaction mechanism: (1) condensation of aniline with paraformaldehyde forms imine intermediate **68**; (2) electrophilic addition of **68** to aniline generates diamine adduct **69**; (3) condensation with paraformaldehyde constructs the nitrogen bridge at the amine sites of **69**; and (4) a final electrophilic addition completes assembly of the TB scaffold. These attributes make TB derivatives promising candidates for supramolecular chemistry and functional materials development.

**Figure 12:**
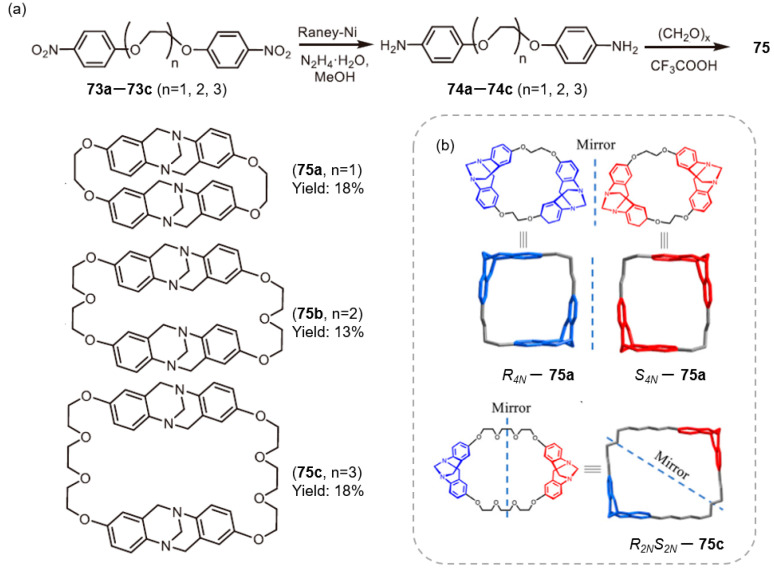
(**a**) Synthesis of ethylene glycol-link-TB macrocyclic path diagram; (**b**) Schematic diagram of the mirror relationship between ethylene glycol-linked TB macrocyclic. Copyright © 2023 Published by Elsevier B.V. on behalf of Chinese Chemical Society and Institute of Materia Medica, Chinese Academy of Medical Sciences.

Wang and co-workers reported the synthesis of TB-derived macrocycles (Figure 12a) [79]. To address the longstanding challenges of low yields and poor stereoselectivity in TB scaffold construction, the authors employed ethylene glycol-bridged phenylenediamine precursors of varying chain lengths to synthesize macrocycles **75a**–**75c** via a one-step TB reaction. The length of the ethylene glycol linker was found to play a critical role in modulating macrocyclic chirality: (1) short chains (**75a**) exclusively yielded homochiral R_4_N/S_4_N isomers; (2) medium chains (**75b**) produced a mixture of homochiral (R_4_N/S_4_N) and heterochiral (R_2_NS_2_N) isomers; and (3) long chains (**75c**) generated only heterochiral configurations (Figure 12b). Although the mechanistic basis for this chain length–chirality relationship remains to be fully elucidated, this work provides a valuable experimental foundation for the stereoselective synthesis of TB-based macrocycles and advances the development of chiral supramolecular systems.

**Figure 13:**
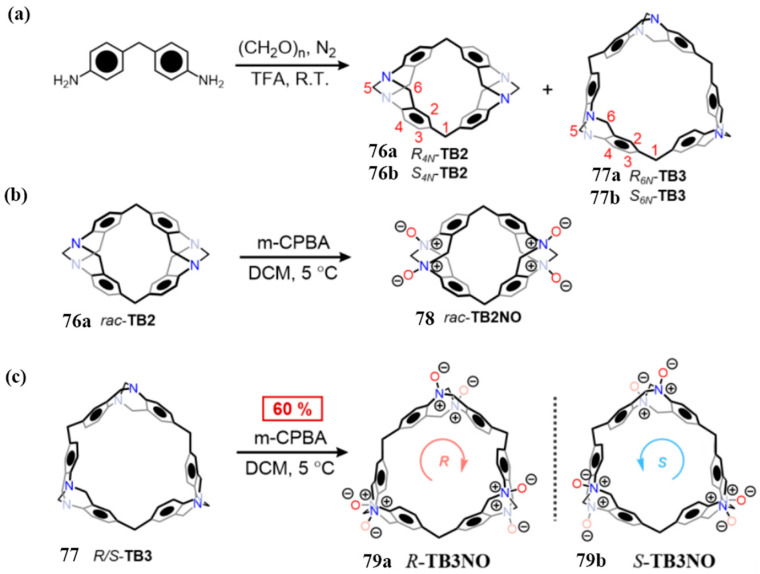
(**a**) Synthesis of macrocycle **76**–**77** and (**b**,**c**) nitrogen oxygen macrocycle **78**–**79** path diagram. Copyright © 2024 Wiley-VCH GmbH.

Yang and co-workers reported a synthetic advancement in the construction of Tröger base (TB)-derived macrocycles [53]. Utilizing diphenylamine precursors, compounds **76** and **77** were synthesized via single-step TB formation reactions (Figure 13a), establishing the TB_n_ (*n* = 2–3) macrocyclic series. The resulting methylene-bridged TB_2_ and TB_3_ macrocycles were obtained as racemic mixtures, each consisting of homochiral configurations (R_4_N/S_4_N and R_6_N/S_6_N, respectively). Enantiopure forms were successfully isolated via chiral HPLC. Subsequent oxidation of the resolved enantiomers with m-CPBA afforded water-soluble N-oxides **78** and **79**, with complete retention of the original TB chirality (Figure 13b,c).

**Figure 14:**
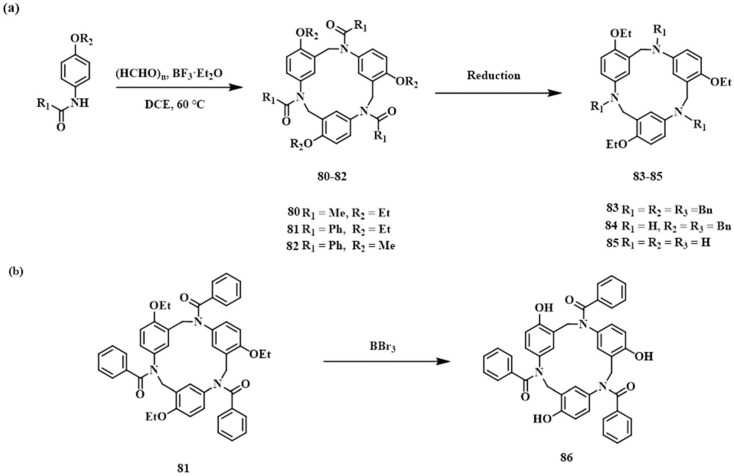
(**a**) Synthesis of Phenacetin[3]arenes **80**–**85**; (**b**) derivatization of the alkoxy end group of **86**.

More recently, Yang and co-workers developed a one-pot synthesis of phenacetin-derived[3]arene macrocycle **80** via a Mannich-type condensation (Figure 14) [54]. This acid-catalyzed reaction between phenacetin and paraformaldehyde establishes a novel cyclization pathway, forming methylene bridges between aromatic amines and aryl carbons (N_Am_-CH_2_-C_Ar_). Compound **80** exhibits unique structural features, tunable chemical functionality, and molecular recognition capabilities. Phenacetin, a commercially available p-aminophenol derivative and widely used antipyretic/analgesic, served as the starting material. Under BF_3_·OEt_2_ catalysis, the one-pot reaction afforded **80**, with uncyclized oligomers as the primary byproducts. Homologs of phenacetin underwent analogous cyclization to yield compounds **81**–**82**, enabling gram-scale production. Subsequent reduction, dealkylation, or hydrolysis of **80** afforded derivatives **83**–**85**, offering multiple sites for further functionalization.

## 3. Applications of N-Containing Macrocyclic Arenes

### 3.1. Molecular Recognition

#### 3.1.1. Recognition of Cationic Guests

**Figure 15:**
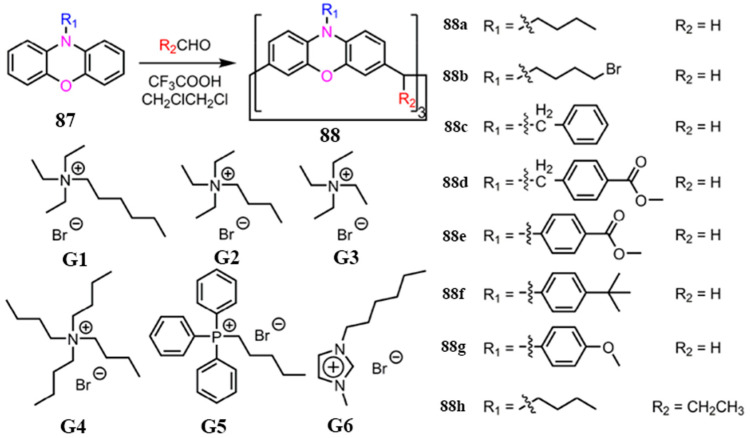
The synthetic route of calix[3]phenoxazine **88a**–**88h** and the chemical structures of guests **G1**–**G6**. Copyright © 2023, American Chemical Society.

Nitrogen-containing macrocyclic arenes exhibit selective recognition toward electron-deficient guests, particularly cations, due to their electron-rich aromatic cavities. A representative example is provided by the Motuo and co-workers: their conical calix[3]phenoxazine **88a** (Figure 15) forms a stable 1:1 complex with the cationic guest G1, with a binding constant of (1.66 ± 0.15) × 10^3^ M^−1^. This result highlights the superior cation-binding affinity of such macrocyclic systems [80].

**Figure 16:**
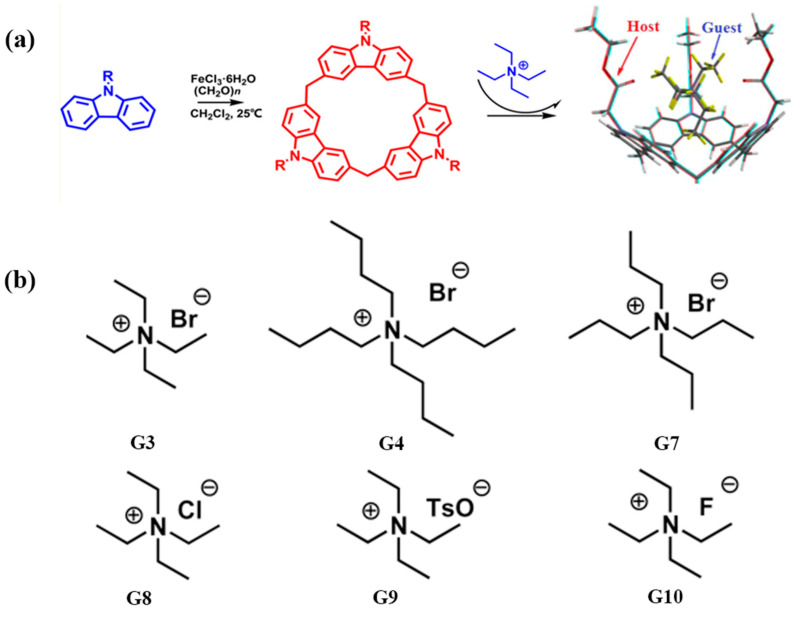
(**a**) synthesis of calix[3]carbazole compounds; (**b**) guest of the test. Copyright © 2016, American Chemical Society.

Calix[3]carbazole **41** (Figure 16) exhibits distinctive molecular recognition behavior. Its enlarged, semi-open, π-electron-rich cavity selectively binds tetraethylammonium (TEA^+^) through cation–π interactions, affording stable 1:1 complexes. In contrast, its binding affinity for larger guests such as tetrapropyl- and tetrabutylammonium is significantly diminished, highlighting its stringent size selectivity [57].

**Figure 17:**
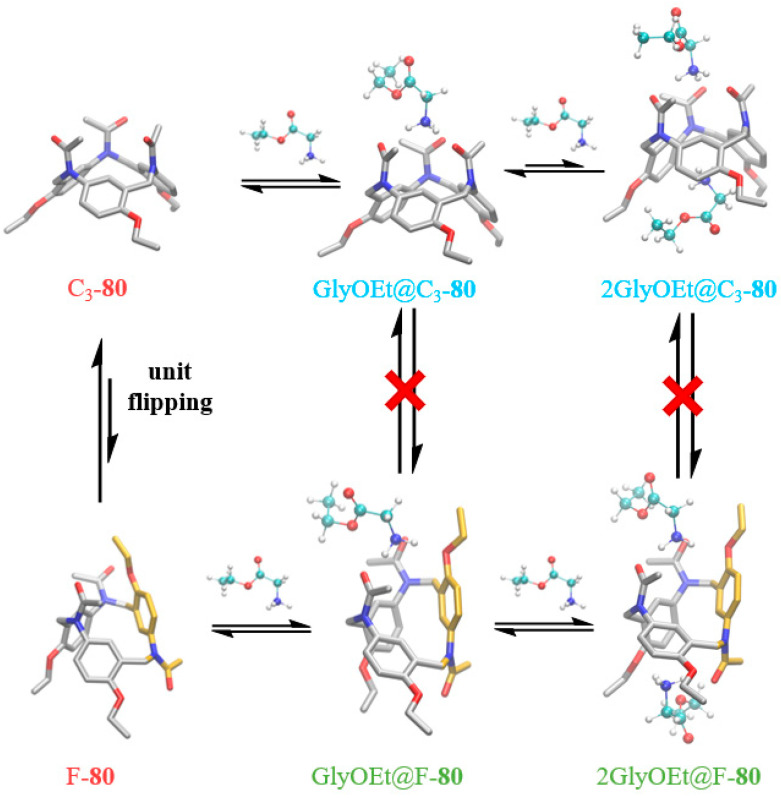
The equilibrium diagrams of **C_3_-80** and **F-80** single host, 1:1, and 1:2 host–guest complexes. Copyright *© 2025 Wiley-VCH GmbH*.

Phenacetin[3]arene **80** [54] exhibits a pronounced affinity toward primary ammonium salts. Host–guest complexes were characterized via ^1^H NMR, ^13^C NMR, ITC, circular dichroism, and DFT calculations (Figure 17). Compound **80** forms 1:2 complexes, wherein the initial 1:1 association is governed by triple hydrogen bonding between the three carbonyl groups of **80** and the NH groups of the guest, yielding binding constants (K_1_) on the order of 10^5^~10^6^ M^−1^, as determined by ITC. The resulting 1:2 complex displays allosteric behavior: Rim A stabilizes the guest through two C=O···H–N hydrogen bonds and one NH···π interaction, whereas rim B engages one C=O···H–N bond and two NH···π interactions. The stepwise binding constant for the second guest (K_2_) is approximately 10^3^ M^−1^.

#### 3.1.2. Enantioselective Recognition of Chiral Molecules

**Figure 18:**
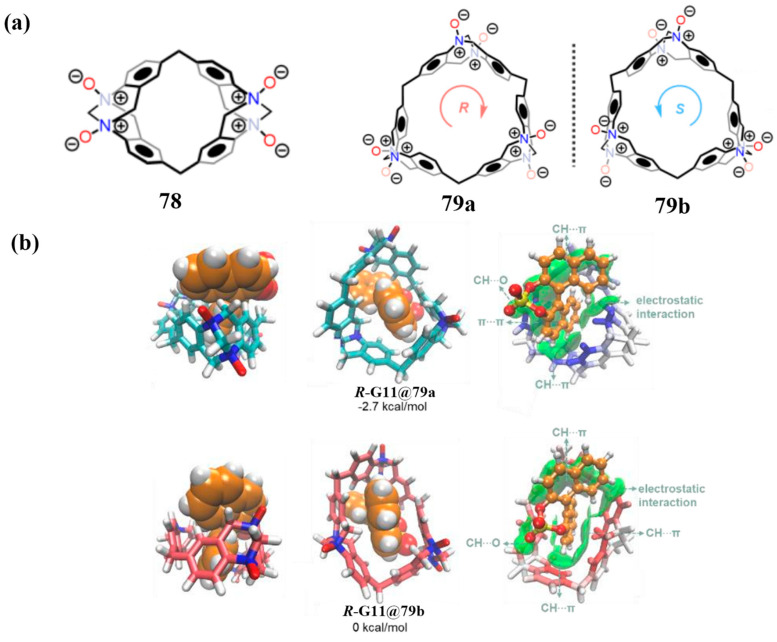
(**a**) Structure diagram of nitrogen oxide macrocycle **78**–**79**; (**b**) the binding diagram of naphthyl phosphate **G11**. Copyright © 2024 Wiley-VCH GmbH.

Chiral recognition—a fundamental molecular recognition process in biological systems [14,15,29,81,82,83,84,85,86,87,88,89]—holds significant practical value in asymmetric catalysis [90,91], chiral drug development [92], and the design of functional materials [93]. Macrocyclic arenes serve as ideal platforms for investigating stereoselectivity due to their tunable cavity geometries and well-defined stereochemical environments. Recent advances in macrocyclic arene receptors with rigid frameworks or internally modified cavities have yielded systems with exceptional chiral recognition capabilities. A notable example is the water-soluble nitrogen-bridged macrocycles **78**–**79** (Figure 18a), reported by Yang and co-workers. These macrocycles not only preserve the chiral configuration of the tert-butyl substituents but also exhibit pronounced enantioselectivity toward chiral guests in aqueous media, attributed to their structural rigidity. Remarkably, the selectivity coefficient toward naphthyl phosphate guest **G11** reaches 41.0—the highest reported for macrocyclic arenes to date (Figure 18b). These results offer valuable guidance for the development of next-generation chiral separation materials and asymmetric catalysts [53].

### 3.2. Adsorption and Separation

**Figure 19:**
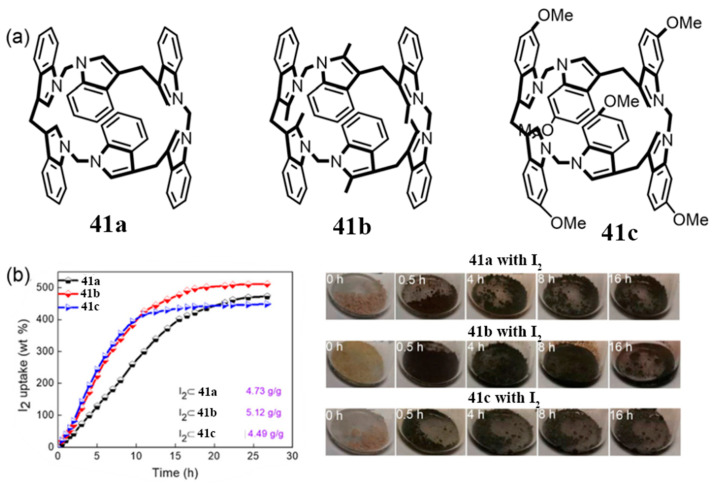
(**a**) Structures of bisindolo[3]arene **41a**–**41c**; (**b**) time-dependent I_2_ vapor adsorption curve. Copyright © 2021 Chinese Chemical Society.

Iodine is a common nuclear contaminant of significant concern. The radioisotope ^129^I, characterized by its long half-life (1.57 × 10^7^ years), high environmental mobility, and toxicity [94], poses persistent ecological risks. In contrast, ^131^I, though short-lived (t_1/2_ = 8.02 days), presents serious radiological hazards due to its propensity to accumulate in the thyroid during human metabolism [95,96]. Consequently, efficient radioactive iodine capture is vital, particularly in the context of radiological emergencies. Yang and co-workers conducted a systematic study on iodine vapor adsorption using bisindole[3]arene derivatives **41a**–**41c** (Figure 19). These macrocyclic powders exhibited pronounced chromogenic responses upon iodine uptake. Among them, compound **41c** showed the highest adsorption capacity (5.12 g/g), outperforming **41a** (4.49 g/g) and **41b** (4.73 g/g). Mechanistic investigations revealed that these initially non-porous materials undergo substantial structural reorganization during adsorption, with the exceptional iodine capture efficiency attributed to intra- and intermolecular synergistic effects. Furthermore, fluorescence quenching observed in 41c confirms charge transfer interactions between the indole moieties and iodine. These findings provide key molecular design insights for the development of next-generation iodine capture materials [67].

**Figure 20:**
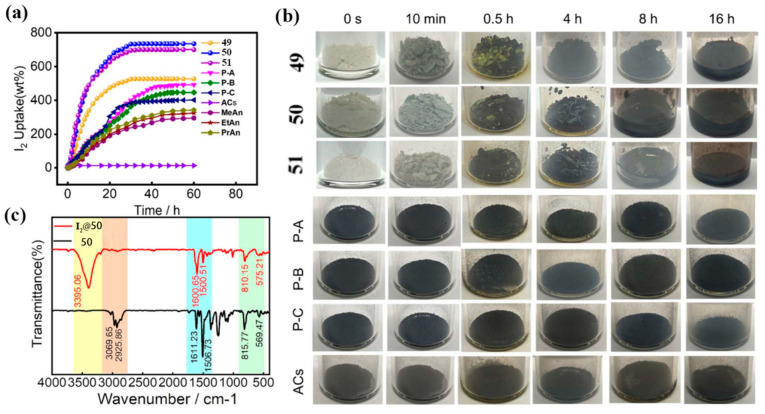
(**a**) at 348.15 K, the I_2_ vapor absorption rate of **49**−**51**, Ans, P-Ns, and ACs versus time curves; (**b**) photos of **49**–**51**, P-Ns, and ACs absorbing I_2_ vapor at different time intervals. (**c**) FT-IR spectra of **50** and I_2_-**50**.

In addition, macrocyclic diphenylamine **49**–**51** exhibited outstanding iodine adsorption performance (Figure 20), with a maximum uptake capacity of 7.35 g/g in both gas-phase and solution-phase environments [69]. The study revealed a distinctive reverse dissolution–adsorption mechanism: upon exposure to iodine, the crystalline forms of **49**–**51** undergo a phase transition to amorphous states, substantially enhancing their iodine-loading capacity. Notably, iodine can be reversibly desorbed through thermal treatment or exposure to organic solvents, and the materials retain stable adsorption performance over multiple cycles. Despite their limited apparent specific surface areas, the electron-rich nitrogen-containing frameworks of **49**–**51** facilitate stable iodine capture via efficient electron transfer. These findings offer a novel strategy for the molecular design of advanced adsorption materials.

Zeng and co-workers designed novel electron-deficient pillar[6]arene derivatives featuring a biphenyltetracarboxylic diimide-extended structure that forms a nanoscale electron-deficient cavity [97]. This macrocycle demonstrated exceptional separation performance as a gas chromatography stationary phase, efficiently resolving benzene/cyclohexane and toluene/methylcyclohexane mixtures. Compared to conventional capillary columns, this packed column offers distinct advantages: simplified preparation, reduced column length, lower separation temperatures (e.g., 40 °C), and significantly shorter retention times. These properties provide a promising alternative for chromatographic separation technologies.

### 3.3. Novel Photophysical Properties

**Figure 21:**
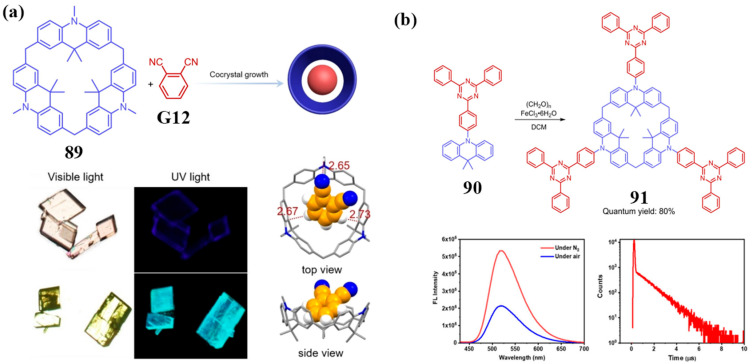
(**a**) Preparation and photos of **89** and **G12** co-crystals; (**b**) S-triphenyltriazine-derived calix[3]acridine **91**; the PL spectra of **91** before and after nitrogen purge and the transient PL attenuation curve of **91** in toluene under saturated nitrogen were studied. Copyright © 2022 Wiley-VCH GmbH.

Significant progress has recently been made in the development of luminescent materials based on novel N-containing macrocyclic arenes, particularly within the realm of thermally activated delayed fluorescence (TADF) systems. Chen and co-workers pioneered a supramolecular TADF (STADF) strategy [98], employing host–guest charge transfer between electron-donating calix[3]acridine **89** and electron-accepting 1,2-dicyanobenzene (**G12**) to achieve efficient STADF emission (Figure 21a). This co-crystal system achieves a remarkable PLQY of 70%—the highest reported for intermolecular donor–acceptor TADF materials. Building on this work, the team further developed calix[3]acridine 91 (Figure 21b), which possesses intrinsic TADF properties by incorporating acridine donor and s-triphenyltriazine acceptor units [99]. Compound **91** exhibits an exceptionally small ΔE_ST_ (12 meV) and a high solution PLQY (80% in toluene), demonstrating outstanding potential as an efficient TADF emitter.

**Figure 22:**
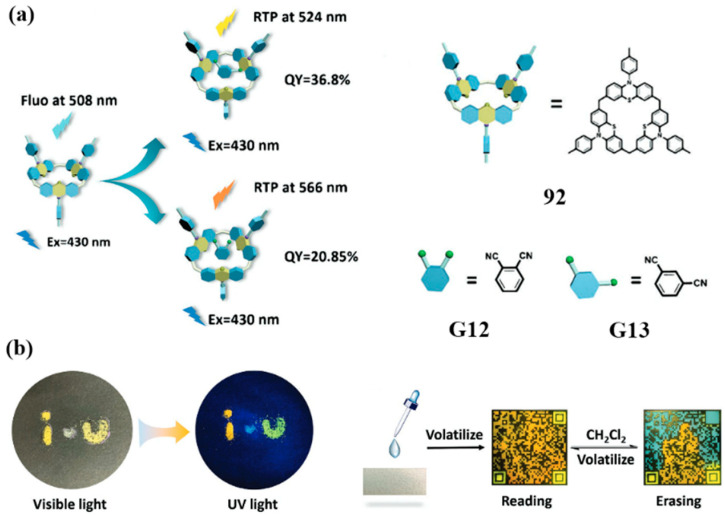
(**a**) The schematics of RTP emission of selectively activated **G12**@**92** and **G13**@**92** are shown; (**b**) photographs of **92**, **G12**@**92,** and **G13**@**92** solid powders under visible light and 365 nm UV light, as well as phosphorescent QR codes and solvent-responsive information storage/encryption. Copyright © 2023 Wiley-VCH GmbH.

Liu and co-workers recently developed an amorphous supramolecular system comprising calix[3]phenothiazine **92** and dicyanobenzene derivatives **G12/G13** (Figure 22a), exhibiting unique organic room-temperature phosphorescence (RTP) properties [100]. These complexes demonstrate unprecedented RTP behavior arising solely from host–guest complexes. Upon 430 nm excitation, C–H···π interactions facilitate the formation of dense supramolecular networks, resulting in phosphorescence emissions at 566 nm (**92/G12**) and 524 nm (**92/G13**). In contrast, the pristine crystals of **92** display only fluorescence at 508 nm. Mechanistic investigations revealed that **G12** and **G13** serve as “molecular rivets,” stabilizing the conformation of **92** and restricting molecular vibrations, thereby reducing non-radiative decay pathways. This structural stabilization leads to high phosphorescence quantum yields ranging from 20.8% to 36.8%. Capitalizing on this guest-activated RTP phenomenon, the authors further demonstrated practical applications, including multicolor quick-response (QR) codes and information encryption (Figure 22b).

**Figure 23:**
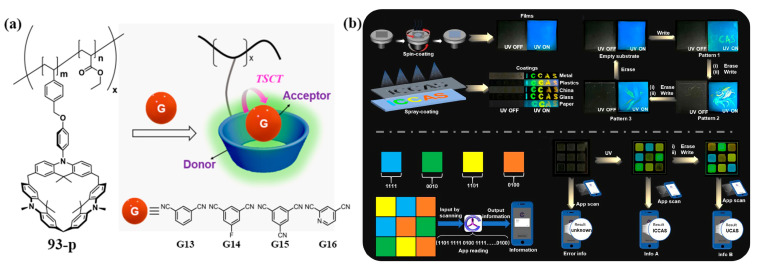
(**a**) The construction of TADF polymer materials based on **93-P**; (**b**) a schematic diagram of the application of the polymer material in advanced information encryption. Copyright © 2024 Chinese Chemical Society.

Recently, Chen and co-workers developed novel dual-emission TADF polymer systems by solution-processing calix[3]acridine-functionalized polymer **93-P** with electron-accepting guests **G13**–**G16** of varying electron affinity (Figure 23a) [101]. The TADF properties originate from through-space charge transfer (TSCT) between the macrocyclic donor in the polymer and electron-accepting guests. Precise modulation of guest electron-accepting strength enables tunable emission colors and achieves photoluminescence quantum yields up to 40%. These materials exhibit superior processability, tunable luminescence, and excellent mechanical stability. Leveraging these advantages, the team applied the systems to information security, developing substrate-independent anti-counterfeiting tags and smartphone-readable QR codes (Figure 23b). This provides novel solutions for advanced information encryption.

## 4. Conclusions and Perspectives

Recent advances in macrocyclic arenes research have significantly advanced supramolecular chemistry [68]. In particular, nitrogen-incorporated macrocycles have garnered broad interdisciplinary interest due to their distinctive host–guest recognition capabilities [53,54]. This perspective categorizes recent synthetic strategies and applications of N-containing macrocyclic arenes based on their macrocyclization approaches: (1) nitrogen-bridging macrocycles and (2) non-nitrogen-bridging macrocycles. These developments not only expand the architectural diversity of macrocyclic arenes but also establish new design paradigms relevant to chemistry, biology, and materials science.

Despite notable progress in the chemistry of macrocyclic arenes [16], the design, synthesis, and application of structurally novel N-containing macrocyclic arenes continue to present fundamental challenges. Owing to their unique structural features, these macrocycles hold great promise for the development of N-containing functional molecules. One-pot condensation of polycyclic aromatic or heteroaromatic units offers a promising route to constructing N-containing macrocyclic arenes due to its synthetic efficiency. However, the presence of active hydrogens in primary and secondary amines often leads to side reactions, complicating the reaction landscape. Tertiary amines are frequently employed as macrocyclization units. Furthermore, the judicious selection of building blocks, in combination with the use of directing groups, significantly enhances synthetic efficiency. For unmodified polycyclic aromatic systems, post-synthetic modification of macrocyclic frameworks provides an alternative and complementary synthetic strategy.

Macrocyclic arenes serve as key supramolecular functional materials owing to their tunable cavity dimensions and superior electronic properties. Through rational molecular design, these macrocycles enable highly efficient cation recognition, offering promising applications in chiral separations, environmental remediation, and optoelectronic devices. Nonetheless, several critical challenges must be addressed to achieve practical, high-performance materials. In the context of chiral separations, macrocyclic arene-based platforms offer promising solutions for the enantioselective interaction of pharmaceuticals and bioactive molecules. While successful chiral recognition examples have been reported, systems that simultaneously exhibit high enantioselectivity, strong affinity, and long-term cycling stability remain limited. For environmental applications, these macrocycles demonstrate strong affinity for aqueous organic pollutants and heavy metal ions via specific host–guest interactions. However, their implementation in real-world wastewater treatment requires further validation. As optoelectronic materials, macrocyclic arenes suppress non-radiative decay through their rigid skeletal frameworks, resulting in enhanced fluorescence quantum yields. Moreover, host–guest complexation modulates inter- and intramolecular charge transfer processes, allowing precise control over luminescence color and enabling long-lived emission (e.g., RTP). These properties support a wide range of applications in sensors, organic light-emitting diodes (OLEDs), and advanced anti-counterfeiting technologies. Nevertheless, the field still faces major challenges in conducting systematic mechanistic studies and improving material stability.

Although the practicality of macrocyclic aromatic hydrocarbons has been proven, their functionalization, scalable synthesis, and conversion applications still face significant obstacles. Future research should combine computational simulation, rational molecular design, and multidisciplinary approaches to accelerate the transition from basic research to practical applications. This rapidly evolving field offers abundant opportunities at the interfaces of supramolecular chemistry, life sciences, and advanced materials. Overcoming existing technical limitations and harnessing the full potential of macrocyclic arenes will pave the way for transformative advances in both fundamental science and technological innovation.

## Data Availability

Not applicable.
